# Changes in surgicaL behaviOrs dUring the CoviD-19 pandemic. The SICE CLOUD19 Study

**DOI:** 10.1007/s13304-021-01010-w

**Published:** 2021-03-03

**Authors:** Umberto Bracale, Mauro Podda, Simone Castiglioni, Roberto Peltrini, Alberto Sartori, Alberto Arezzo, Francesco Corcione, Ferdinando Agresta, the CLOUD-19 Collaborative Group, Adelmo Antonucci, Claudia Zanframundo, Fabio Cavallo, Giorgio Mazzarolo, Antonio Agrusa, Giuseppe Di Buono, Luca Aldrighetti, Guido Fiorentini, Alessandro Lucianetti, Stefano Magnone, Sergio Alfieri, Fausto Rosa, Donato F. Altomare, Arcangelo Picciariello, Amilcare Parisi, Antonio Di Cintio, Marco Francesco Amisano, Francesca Cravero, Michele Ammendola, Giorgio Ammerata, Alessandro Anastasi, Giuseppe Canonico, Andra Gattolin, Elisabetta Travaglio, Andrea Sartori, Massimiliano De Palma, Pierluigi Angelini, Francesco Galante, Angelo Benevento, Stefano Rausei, Angelo Serao, Francesca Abbatini, Mario Annecchiarico, Antonio Varricchio, Valerio Annessi, David Tumiati, Alfredo Annicchiarico, Antonello Mirabella, Marco V. Marino, Antonino Spinelli, Antonio Braun, Hong Tham Santi, Lucia Romano, Michele Antoniutti, Mariano Fortunato Armellino, Giulio Argenio, Augusto Verzelli, Andrea Budassi, Gianluca Baiocchi, Marie Sophie Alfano, Alessandro Balani, Marco Barone, Gianandrea Baldazzi, Diletta Cassini, Ruben Carlo Balzarotti Canger, Gianpietro Zabbialini, Andrea Belli, Francesco Izzo, Franco Bertolino, Marco Brunetti, Francesco Bianco, Antonio Cappiello, Luigi Boccia, Bernardo Boffi, Federico Perna, Stefano Bonilauri, Giuseppe Frazzetta, Pierpaolo Bordoni, Francesco Fleres, Felice Borghi, Giorgio Giraudo, Vincenzo Bottino, Alfonso Canfora, Fabrizio Briganti Piccoli, Luca Calligaris, Bruno Nipote, Aniello Gennaro Nasti, Andrea Bufalari, Francesca Bettarini, Massimo Buononato, Marco Greco, Pietro Giorgio Calò, Fabio Medas, Eugenia Cardamone, Pasquale Castaldo, Massimo Carlini, Domenico Spoletini, Carlo De Nisco, Fabio Pulighe, Carlo V. Feo, Nicolò Fabbri, Carmine Antropoli, Fabrizio Foroni, Maurizio Carnazza, Salvatore Ragazzi, Elisa Cassinotti, Luigi Boni, Fausto Catena, Mario Giuffrida, Gennaro Perrone, Christian Ccotsoglou, Stefano Granieri, Graziano Ceccarelli, Walter Bugiantella, Carla Cedolini, Luca Seriau, Maurizio Cesari, Alessandro Contine, Osvaldo Chiara, Stefania Cimbanassi, Eugenio Cocozza, Mattia Berselli, Corrado Fantini, Renato Costi, Lorenzo Casali, Andrea Morini, Francesco Crafa, Serafino Vanela, Giuseppe Currò, Vincenzo Orsini, Corrado Da Lio, Mario Biral, Piergiorgio Danelli, Claudio Guerci, Dario Scala, Graziella Marino, Luciano De Carlis, Andrea Lauterio, Donato De Giorgi, Gianluca Sciannamea, Nicolo De Manzini, Pasquale Losurdo, Maurizio De Palma, Nicola Sangiuliano, Maurizio Degiuli, Franco Caterina, Paolo Del Rio, Elena Bonati, Stefano Di Lernia, Marco Vittorio Rossi Ardizzone, Salomone Di Saverio, Caterina Franchi, Beatrice Di Venere, Rosanna Miglio, Diego Cuccurullo, Carlo Sagnelli, Ludovico Docimo, Salvatore Tolone, Mauro Longoni, Giuseppe Faillace, Fabio Rondelli, Francesca Pennetti Pennella, Vincenzo Colucci, Teresa Carfora, Irnerio Angelo Muttillo, Biagio Picardi, Rossi Stefano, Roberto Campagnacci, Angela Maurizi, Fausto Tricarico, Marco Montagna, Elio Amedeo, Michela C. Scollica, Enrico Lauro, Ernesto Laterza, Enrico Molinari, G. Berta, Dario Bono, Massimiliano Fabozzi, Mafalda Romano, Enzo Facci, Dario Parini, Roberto Farfaglia, Valeria Arizzi, Marco Farsi, Egidio Miranda, Landino Fei, Giordano Flavio, Felice Pirozzi, Antonio Sciuto, Alessandro Ferrero, Marco Palisi, Marco Filauro, Andrea Barberis, Antonio Azzinnaro, Valentino Fiscon, Silvia Vigna, Michele D’ambra, Emanuele Pontecorvi, Gabriele Anania, Cristina Bombardini, Gennaro Galizia, Annamaria Auricchio, Francesca Cardella, Michele Genna, Sergio Gentilli, Nikaj Herald, Giampaolo Castagnoli, Alberto Bartoli, Luca Gianotti, Mattia Garancini, Giovanni Bellanova, Paola Palazzo, Giovanni De Palma, Marco Milone, Giovanni Ferrari, Carmelo Magistro, Antonio Giuliani, Giuseppe Di Natale, Giuseppe Brisinda, Giuseppe Cavallaro, Giuseppe Sammarco, Gaetano Gallo, Orlando Goletti, Daniele Macchini, Vincenzo Greco, Vincenzo Amoroso, Gianluca Guercioni, Michele Benedetti, Guglielmo Guzzo, Francesco Pata, Ildo Scandroglio, Francesco Roscio, Elio Jovine, Raffaele Lombardi, Francesco La Rocca, Francesca Di Capua, Carmine Lanci, Renzo Leli, Andrea Borasi, Pasquale Lepiane, Andrea Balla, Edoardo Liberatore, Luca Morelli, Gregorio Di Franco, Andrea Lucchi, Laura Vittori, Luigi Bonavina, Emanuele Asti, Dario Maggioni, Gerosa Martino, Giuseppe Manca, Antonella Delvecchio, Manfredo Tedesco, Denise Gambardella, Salvatore Marafioti, Maria Luisa De Marco, Marco Azzola Guicciardi, Massimo Motta, Marco Calgaro, Vincenzo Adamo, Mario Guerrieri, Pietro Coletta, Monica Ortenzi, Gennaro Martines, Giuliano Lantone, Mario Martinotti, Giuseppe Fassardi, Maurizio Castriconi, Simone Squillante, Maurizio De Luca, Maurizio Pavanello, Carlo Di Marco, Maurizio Ronconi, Silvia Casiraghi, Vincenzo Mazzaferro, Carlo Battiston, Michele Perrotta, Carmine Ripa, Micheletto Giancarlo, Valerio Panizzo, Paolo Millo, Riccardo Brachet Contul, Valentina Ferraro, Carlo Molino, Enrico Crolla, Gianluigi Moretto, Matilde Bacchion, Mario Morino, Marco Ettore Allaix, Enrico Motterlini, Michele Petracca, Andrea Muratore, Mario Musella, Antonio Vitiello, Bruno Nardo, Veronica Crocco, Giuseppe Navarra, Salvatore Lazzara, Giuseppe Giovanni Navarra, Manuela Cuoghi, Stefano Olmi, Alberto Oldani, Matteo Uccelli, Enrico Opocher, Marco Giovenzana, Paolo De Paolis, Mauro Santarelli, Paolo Delrio, Fabio Carbone, Paolo Pietro Giampaolo Bianchi, Formisano, Patrizio Capelli, Edoardo Baldini, Patrizio Festa, Arianna Mottola, Michele Perrotta, Giovanni Merola, Nicola Perrotta, Marta Celiento, Eraldo Personnettaz, Stefania Muzio, Tommaso Petitti, Antonietta Melchiorre, Micaela Piccoli, Francesca Pecchini, Alice Frontali, Piergiorgio Danelli, Anna Maffioli, Pietro Maida, Pasquale Tammaro, Giusto Pignata, Jacopo Andreuccetti, Vincenzo Pilone, Michele Renzulli, Salvatore Pintaldi, Andrea Pisani Ceretti, Nicolò Maria Mariani, Adolfo Pisanu, Roberto Polastri, Fabio Maiello, Alberto Porcu, Teresa Perra, Felice Mucilli, Mirko Barone, Roberto Troisi, Roberto Montalti, Fabrizio Scognamillo, Daniele Delogu, Raffaele Galleano, Michele Malerba, Raffaele Salfi, Marcello Pisano, Raffaele Sechi, Nicola Cillara, Salvatore Ramuscello, Eugenio De Leo, Enrico Restini, Rocco Tumolo, Pasquale Cianci, Sabino Capuzzolo, Maurizio Rizzo, Alfonso Recordare, Roberto Santoro, Pietro Maria Amodio, Aldo Rocca, Giuseppe Cecere, Raffaele Romito, Luca Portigliotti, Riccardo Rosati, Ugo Elmore, Domenico Russello, Saverio Latteri, Salvatore Maria Costarella, Salvatore Massa, Lorenzo Capasso, Michele Santangelo, Maurizio Sodo, Giuliano Sarro, Umberto Rivolta, Stefano Scabini, Davide Pertile, Federico Selvaggi, Selene Rossi, Francesco Selvaggi, Gianluca Pellino, Gabriele Sganga, Pietro Fransvea, Silvio Testa, Clemente De Rosa, Walter Siquini, Cristian Tranà, Mario Solej, Stefano Bolzon, Enrico Guerra, Marco Stella, Francesco Ferrara, Francesco Stipa, Enrico Stringhi, Andrea Celotti, Lucio Taglietti, Roberto Del Giudice, Carlo Alessandro Talarico, Michele Ruggiero, Giuseppe Tirone, Uberto Fumagalli Romario, Wanda Petz, Valerio Caracino, Valentina Rossetti, Luca Andrea Verza, Fabio Cavallo, Lorenzo Vescovi, Michele Marini, Nereo Vettoretto, Emanuele Botteri, Leonardo Vincenti, Giusy Giannandrea, Tiziana Viora, Lorenzo Maganuco, Paolo Veronesi, Bruno Zani, Giacomo Zanus, Marco Brizzolari, Federico Zanzi, Anna Guariniello, Marco Antonio Zappa, Elisa Galfrascoli, Sandro Zonta, Luigi Oragano, Walter Zuliani, Damiano Chiari

**Affiliations:** 1 Department of General Surgery and Specialties, University Federico II of Naples, Naples, Italy; 2 Department of Emergency Surgery, Policlinico Universitario Di Monserrato, Azienda Ospedaliero-Universitaria Di Cagliari, Cagliari, Italy; 3 Department of Medical, Oral and Biotechnological Sciences, University “G. D’Annunzio” Chieti-Pescara, Chieti, Italy; 4 Department of General, Oncological and Metabolic Surgery, Castelfranco and Montebelluna Hospitals, Treviso, Italy; 5 Department of Surgical Sciences, University of Torino, Torino, Italy; 6 Department of General Surgery, Ospedale Di Vittorio Veneto, ULSS 2, Marca Trevigiana, Italy

**Keywords:** Survey, Laparoscopic surgery, COVID-19, Elective surgery, Emergency surgery

## Abstract

**Background:**

The spread of the SARS-CoV2 virus, which causes COVID-19 disease, profoundly impacted the surgical community. Recommendations have been published to manage patients needing surgery during the COVID-19 pandemic. This survey, under the aegis of the Italian Society of Endoscopic Surgery, aims to analyze how Italian surgeons have changed their practice during the pandemic.

**Methods:**

The authors designed an online survey that was circulated for completion to the Italian departments of general surgery registered in the Italian Ministry of Health database in December 2020. Questions were divided into three sections: hospital organization, screening policies, and safety profile of the surgical operation. The investigation periods were divided into the Italian pandemic phases I (March–May 2020), II (June–September 2020), and III (October–December 2020).

**Results:**

Of 447 invited departments, 226 answered the survey. Most hospitals were treating both COVID-19-positive and -negative patients. The reduction in effective beds dedicated to surgical activity was significant, affecting 59% of the responding units. 12.4% of the respondents in phase I, 2.6% in phase II, and 7.7% in phase III reported that their surgical unit had been closed. 51.4%, 23.5%, and 47.8% of the respondents had at least one colleague reassigned to non-surgical COVID-19 activities during the three phases. There has been a reduction in elective (> 200 procedures: 2.1%, 20.6% and 9.9% in the three phases, respectively) and emergency (< 20 procedures: 43.3%, 27.1%, 36.5% in the three phases, respectively) surgical activity. The use of laparoscopy also had a setback in phase I (25.8% performed less than 20% of elective procedures through laparoscopy). 60.6% of the respondents used a smoke evacuation device during laparoscopy in phase I, 61.6% in phase II, and 64.2% in phase III. Almost all responders (82.8% vs. 93.2% vs. 92.7%) in each analyzed period did not modify or reduce the use of high-energy devices.

**Conclusion:**

This survey offers three faithful snapshots of how the surgical community has reacted to the COVID-19 pandemic during its three phases. The significant reduction in surgical activity indicates that better health policies and more evidence-based guidelines are needed to make up for lost time and surgery not performed during the pandemic.

**Supplementary Information:**

The online version of this article (10.1007/s13304-021-01010-w) contains supplementary material, which is available to authorized users.

## Introduction

The spread of the COVID-19 pandemic has affected 219 countries worldwide, with over 88 million reported cases and over 1.9 million deaths globally [1].

The constant increase in the number of patients requiring treatment for the SARS-CoV2 infection represents a challenge for every national health system and, in some cases, could represent their breaking point. In emergency settings, resources must be concentrated and used rationally to handle the pandemic and continue handling the pre-existing diseases. In this context, most surgical departments have been forced to reschedule their activity, giving priority to urgent/emergent surgical cases and non-deferrable oncological cases [2, 3].

COVID-19 patients may potentially expose healthcare providers to the risk of contamination during surgical and anesthetic procedures [4], and positivity cases and some deaths are already occurring among health workers [5, 6]. In this scenario, the surgical community had been pushed to rapidly understand how to deal with the virus's presence and organize the gradual resumption of surgical activity [7].

Many scientific surgical societies [8–10] have published recommendations on the best practices to be followed in managing suspected or confirmed COVID-19 in surgical patients. In addition to general recommendations for all healthcare workers, such as the adoption of appropriate Personal Protective Equipment (PPE) and screening tests, there are also technical indications that affected the organization of the operating rooms and impacted the surgical technique itself, as some advice about the use of Minimally Invasive Surgery (MIS), the use of smoke evacuator devices (SEDs) and high-energy devices (HEDs) has been released.

Italy has been the first Western country in which the COVID-19 pandemic has spread and the one with the highest number of deaths in Europe. Nine months after the first cases of COVID-19 infection were reported in our country, and during the current second wave of the pandemic, it seems useful to analyze how the surgical community has responded to the pandemic crisis.

With the present national survey, we aim to analyze how the spread of COVID-19 has affected elective and emergency surgical activity in Italy, how the surgical patients' screening policies have changed, and what behaviors Italian surgeons have put in place to protect himself/herself.

## Methods

The steering committee of the CLOUD19 study (UB, SC, MP, AA, AS, FA) promoted, under the aegis of the Italian Society of Endoscopic Surgery, a web-based survey to investigate the changes in surgical behaviors during the COVID-19 pandemic reported by surgeons working in general surgery units across Italy. Participation in the survey remained voluntary, as no incentives were offered to participants. All parts of the study and the present manuscript have been checked and presented according to the E-Surveys Checklist for Reporting Results of Internet (CHERRIES) [11].

### Questionnaire development

The study steering committee developed the questionnaire using remote brainstorming after identifying the questions to include. The technical functionality of the electronic questionnaire was tested before the invitations were sent to the potential participants. Once an agreement was reached, the questionnaire was completed using Google Form (Google LLC, Mountain View, California US).

The questionnaire included 56 questions that assessed the changes in surgical behaviors during the COVID-19 pandemic and consisted of three sections:

- Section 1. Hospital organization: 34 questions
- Section 2. Screening policies: 6 questions
- Section 3. The safety profile of surgical operation: 16 questions

The investigation periods, as reported by the *Istituto Superiore di Sanità* (National Institute of Health) of the Italian Health Ministry [12], were:

- Phase 1: from March to May 2020 (which corresponds to the first pandemic wave in Italy)
- Phase 2: from June to September 2020
- Phase 3: from October to December 2020 (which corresponds to the second pandemic wave in Italy)

Only closed-ended questions were used. The steering committee decided to use ranges of predetermined percentages to allow a more accessible aggregation of the data collected. Responses were single or multiple choice. All questions were set as mandatory fields. The estimated mean time to complete the survey was 20 min.

### Study circulation and data handling

On December 1, 2020, the questionnaire was available online and opened for completion until December 31, 2020.

The link (https://docs.google.com/forms/d/e/1FAIpQLSf0LnqZoUziu5ESCO4DrIgNg0LjE-6SzBLni7BMaQV0cbK4cA/closedform) was circulated through personal email invitations to the chiefs of all the 447 Italian surgical departments registered in the list of the Italian Ministry of Health. The survey was restricted to one delegate per surgical unit, as the study aimed to define the changes in surgical behaviors during the three different phases of the COVID-19 pandemic within the Italian departments of surgery, rather than the attitude of the single surgeon. The link to complete the questionnaire was also always available in the area of the SICE website (https://siceitalia.com), a website dedicated to the dissemination of updates on scientific research regarding MIS and surgical innovations, mainly visited by surgeons with a particular interest in laparoscopic and minimally invasive techniques. Baseline information on respondents and names and locations of surgical units were stored with the questionnaire.

Two members of the steering committee (MP and SC) downloaded the survey results and shared them with the other members to analyze and discuss the data.

### Statistical analysis

Categorical variables were reported using counts and percentages. Data from the survey were compared using 5 × 3 contingency tables and analyzed using the χ2 test. P < 0.050 was considered statistically significant. SPSS® version 22 (IBM, Armonk, New York, USA) was used for the statistical analysis.

## Results

The survey took place between December 1st and 31st. A total of 226 surgical departments, out of the 447 invited, took part in the survey. Respondents come from all 20 Italian regions: 116 (51.2%) were from northern regions, 35 (15.6%) from central regions, and 75 (33.2%) from the South.

**Fig. 1:**
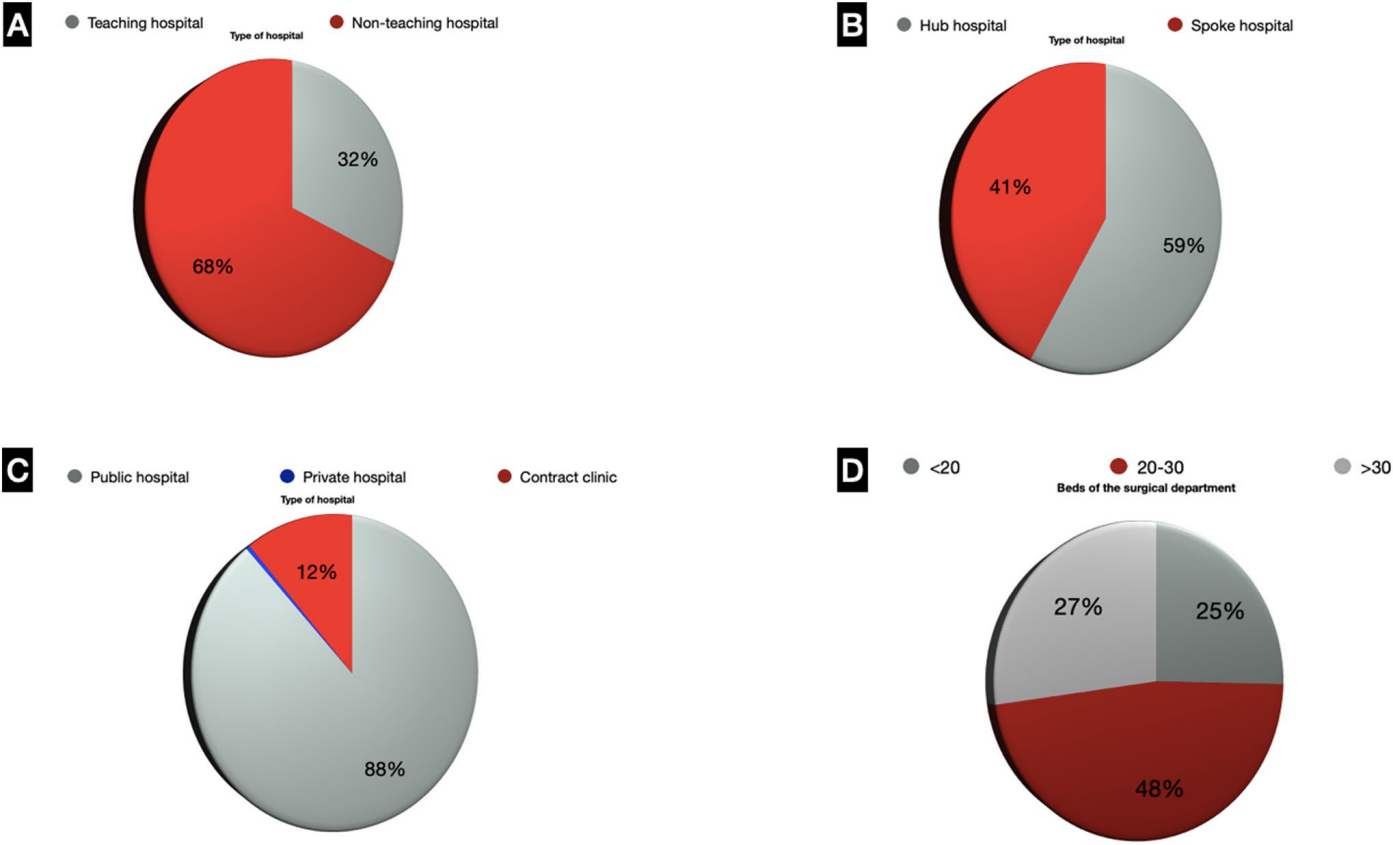
**a** Type of Hospital (Teaching hospital/Non-teaching hospital); **b** Type of Hospital (Hub hospital/Spoke Hospital); **c** Type of Hospital (Public hospital/Private hospital/Contract clinic); **d** Beds of the surgical department

Geographical distribution and hospital characteristics of surveyed surgery departments are shown in Fig. 1 (Supplementary Digital Content_Table1).

## Hospitals' organization in Italy during the three phases of the COVID-19 pandemic

### Configuration of surgical wards

**Fig. 2:**
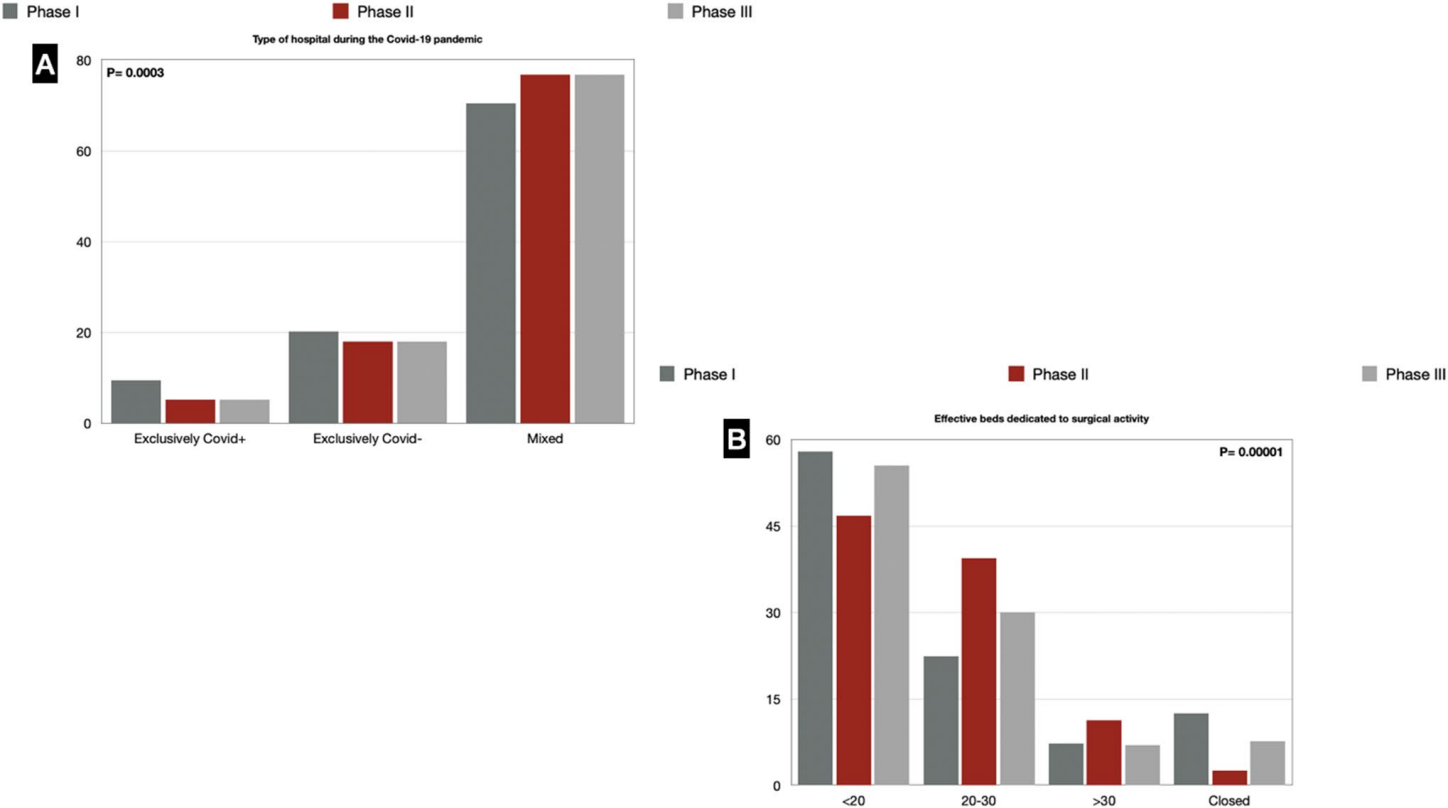
**a** Type of Hospital during the COVID-19 pandemic; **b** Effective beds dedicated to surgical activity during the COVID-19 pandemic

During all three pandemic phases, most Italian hospitals had a mixed configuration in the management of both COVID-19-positive and COVID-19-negative patients, with an increasing trend in such arrangement (70.4% vs. 76.8% vs. 77.4%). During phase I of the pandemic, 9.4% of respondents reported working in a hospital entirely dedicated to COVID-19-positive patients' care. This percentage almost halved in phase II and III (5.2%). The percentage of hospitals dedicated entirely to the care of COVID-19-negative patients decreased through the three study periods (from 20.2% in phase I to 18.0% in phase II and III) (Fig. 2).

### Surgical activity

Almost half of the respondents (47.6%) worked in a surgical department with 20–30 beds in the pre-pandemic era. A reduction in the surgical departments' activity was reported in phase I: 57.9% of the respondents had < 20 effective beds dedicated to the surgical activity, and 11.9% of the respondents stated that their surgical department had been closed. A partial recovery was noted in phase II, with only 2.6% of departments that remained closed. This percentage raised again in phase II when 7.7% of the respondents stated their department's closure. In phase II, 46.8% of the respondents had < 20 effective beds dedicated to the surgical activity, but in phase III, the percentage increased again to 55.4%. The 20–30 beds range decreased to 22.4% in phase I, increased to 39.4% in phase II and decreased to 30.0% in phase III (Fig. 2).

### Elective and emergency surgery

We reported a reduction in terms of surgical activity, both in elective and emergency settings. In phase I, 29.2% of respondents reported having done < 20 elective procedures. There was a gradual return to pre-pandemic conditions in phase II when the range < 20 dropped to 10.3%. Nevertheless, in phase III, surgical activity decreased again, as demonstrated by the fact that the range < 20 rose again to 18.3%. The recovery of surgical activity in phase II was also demonstrated by the fact that the range > 200 procedures rose to 20.6%. In phase III, we report a tendency to slow down again the activity, as the range > 200 has stopped at 9.9%, while the intermediate ranges stabilize.

**Fig. 3:**
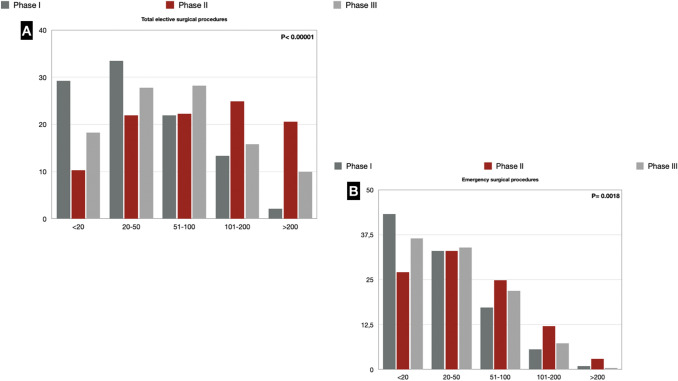
**a** Elective surgical procedure during the COVID-19 pandemic; **b** Emergency surgical procedures during the COVID-19 pandemic

Regarding emergency surgery, 43.3% of respondents stated that they performed < 20 emergency procedures in phase I. There was a resumption of the emergency surgical activity in phase II, as the range < 20 decreased to 27.1%. In phase III, again, there was a decrease in emergency surgical activity (range < 20 = 36.5%) (Fig. 3) (Supplementary Digital Content_Table2).

### Laparoscopic surgery

**Fig. 4:**
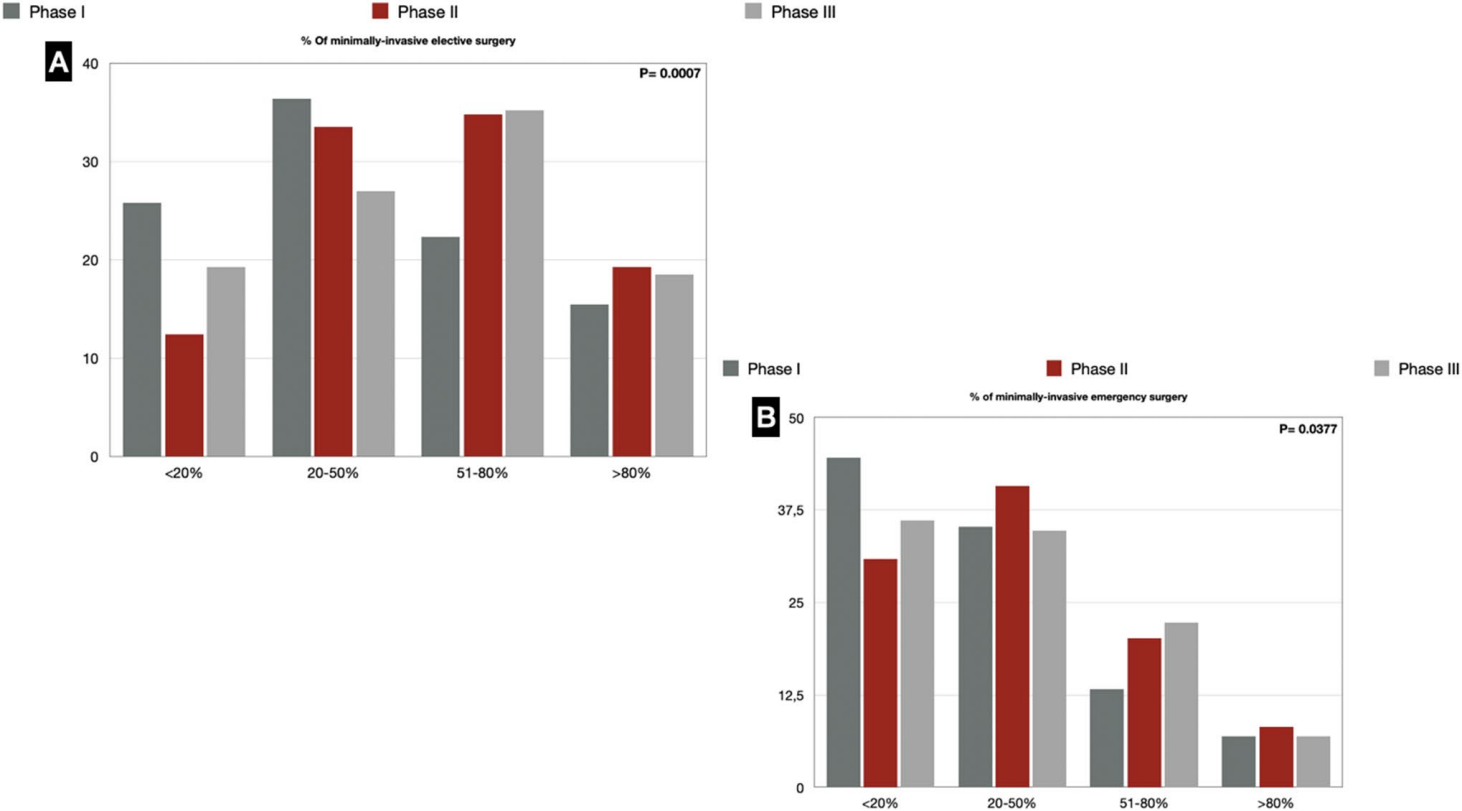
**a** Elective Minimally invasive surgery during the COVID-19 pandemic; **b** Emergency Minimally invasive surgery during the COVID-19 pandemic

Regarding elective laparoscopic procedures, 25.8% of respondents declared that they performed laparoscopy in less than 20% of the total of elective operations in phase I. In Phase II, the range < 20% was halved to 12.4%, only to recover in phase III, with 19.3%. A similar trend emerged for the use of laparoscopy in emergency surgery, with 44.6% of respondents that used laparoscopy in an emergency setting in < 20% of cases. In phase II, 40.7% of answers were in the range 20–50%, whereas, in phase III, 36.1% of respondents performed < 20% of emergency operations with a laparoscopic technique, and 34.7% was in the 20–50% range (Fig. 4) (Supplementary Digital Content_Table3).

### Reassignment of surgical staff

**Fig. 5:**
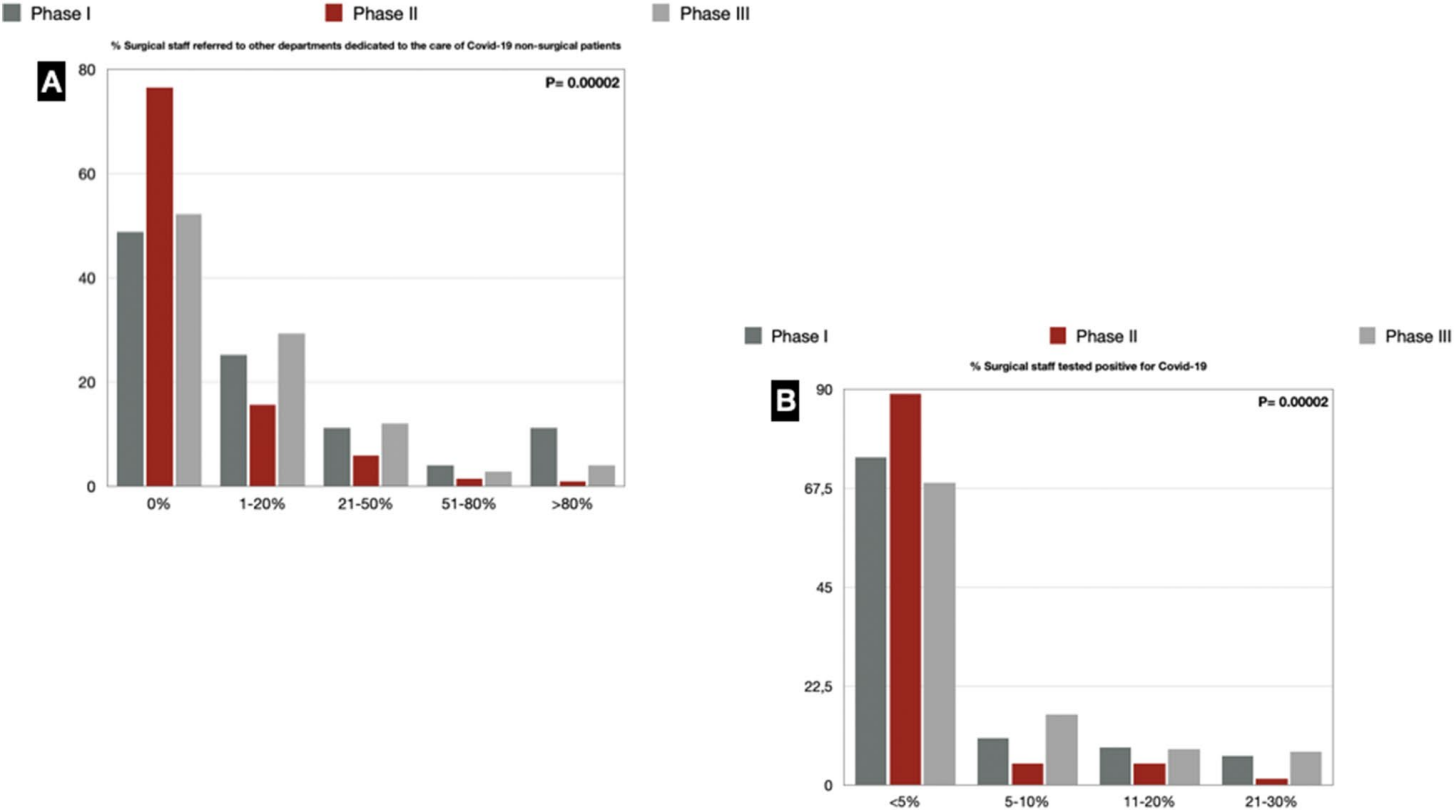
**a** Surgical staff referred to other departments dedicated to COVID-19 non-surgical patients; **b** Surgical staff tested positive for COVID-19

We reported a significant trend towards the reassignment of surgical staff to non-surgical COVID-19 activities in phase I: > 50% of the Italian surgical departments had at least one surgeon assigned to non-surgical activities. The range 1–20% was the one with the highest percentage (25.2%). In phase II, the scenario improved since it was seen that only 23.5% of the surgical departments had a staff member reassigned, with the range 1–20% that dropped to 15.5%. In phase III, the scenario is again worrying, as 48% of departments have at least one surgeon reassigned to non-surgical COVID-19 activities (Fig. 5).

### COVID-19-positive surgeons

The number of COVID-19 cases among surgeons was not negligible: in phase I, 25.6% of the departments had > 5% of surgeons who tested positive for the virus, and in phase III, 31.4% of the departments had > 5% of COVID-19-positive surgeons (Fig. 5) (Supplementary Digital Content_Table4).

### Operated COVID-19-positive patients

**Fig. 6:**
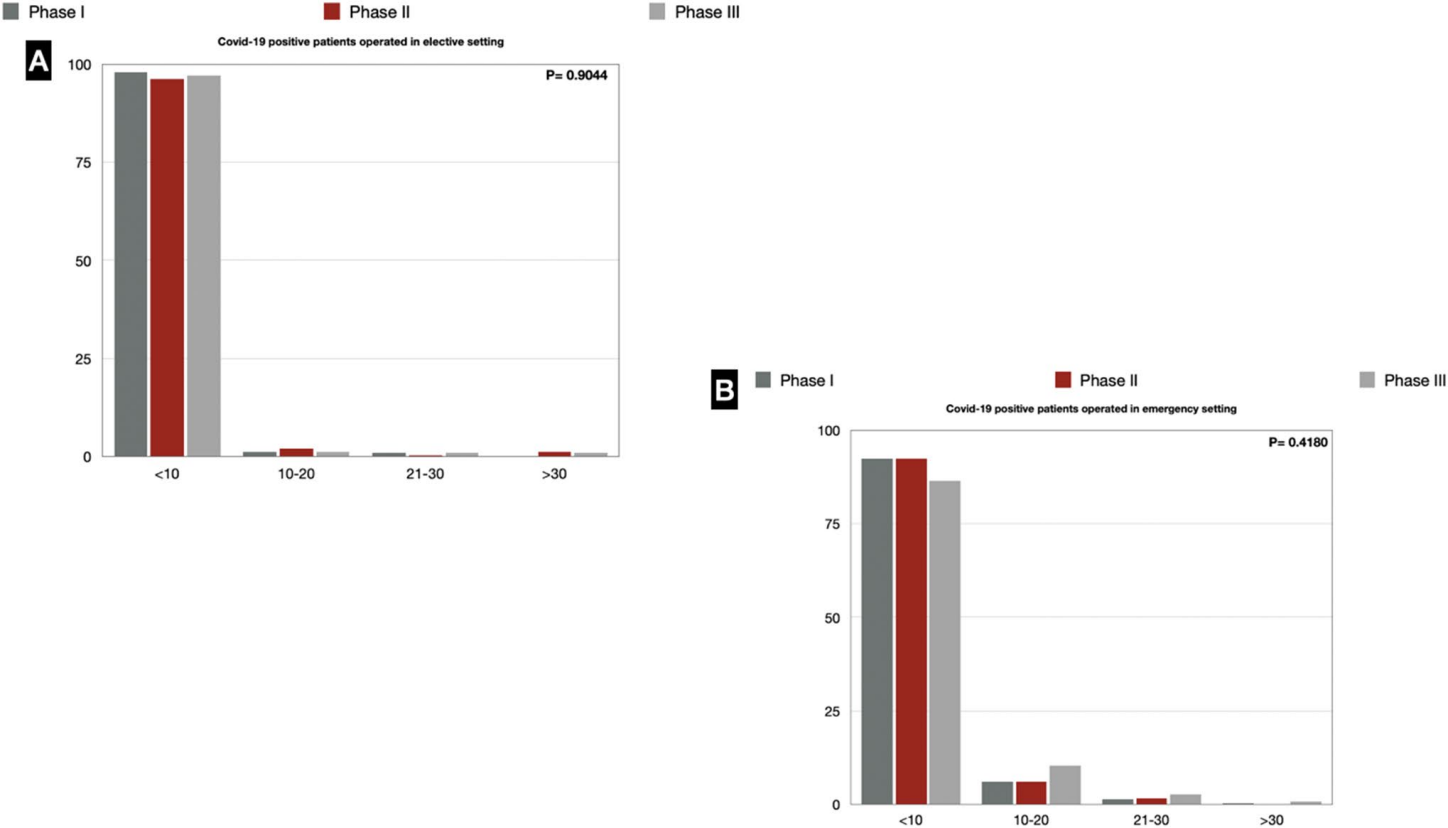
**a** COVID-19-positive patients operated in the elective setting; **b** COVID-19-positive patients operated in emergency setting

Regarding the treatment of COVID-19-positive patients, most of the departments in the three pandemic phases found themselves having to operate < 10 COVID-19-positive patients for emergency surgical disease (92.3% vs. 92.3% vs. 86.3%). A similar scenario was found for elective surgery, as surgery has to be postponed until nasopharyngeal swab for RT-PCR test is negative (Fig. 6).

## Screening policies for SARS-CoV2 in Italy during the three phases of the pandemic

**Fig. 7:**
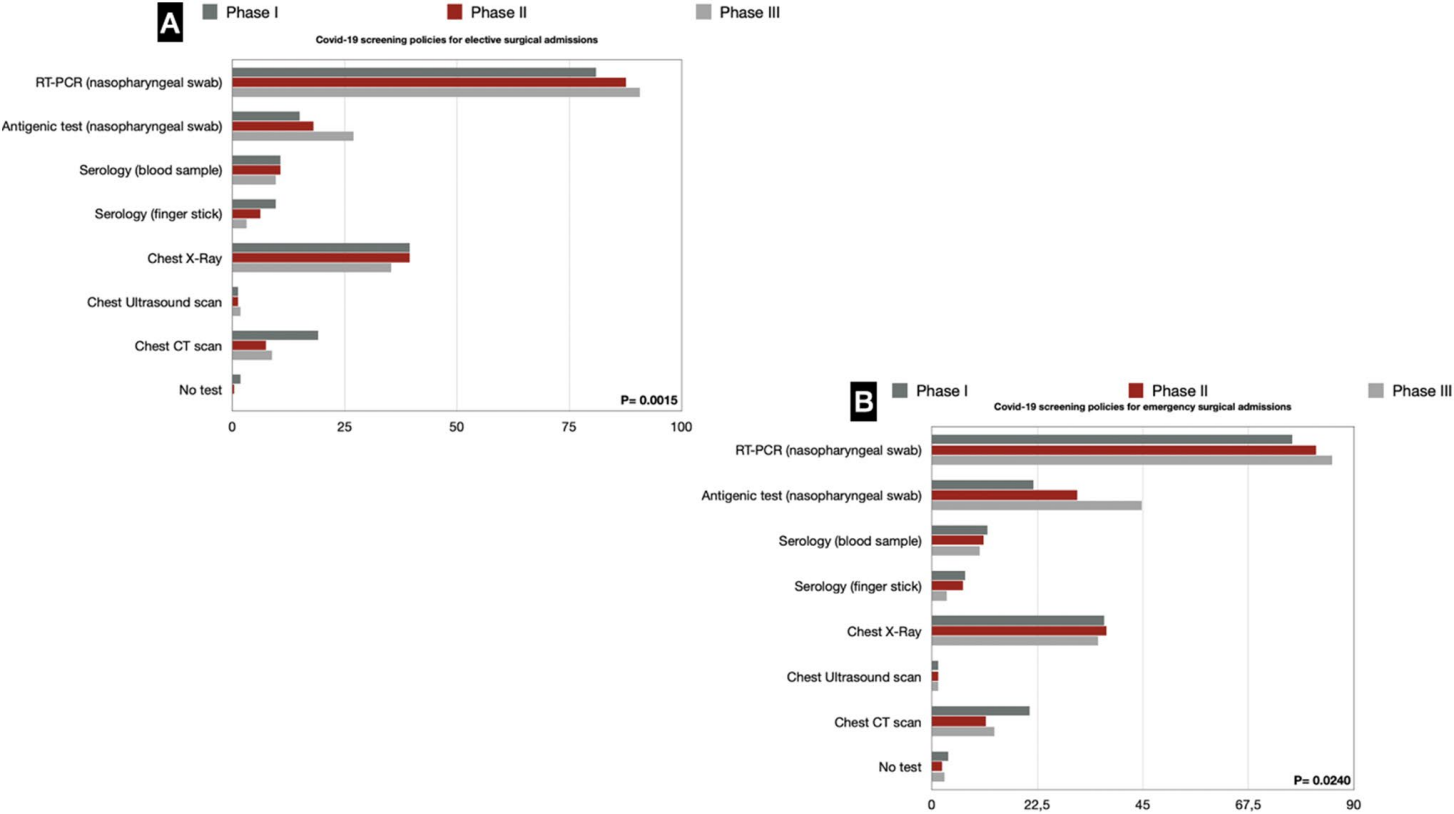
**a** COVID-19 screening policies for elective surgical admissions; **b** COVID-19 screening policies for emergency surgical admissions

Almost all respondents stated that in their hospitals, RT-PCR molecular tests on nasopharyngeal swabs for elective (81%) and emergency (77.9%) surgical patients were routinely used since phase I of the pandemic. Data further improved in phases II and III, reaching 90.7% and 85.4%, respectively. Currently, all elective surgical patients are swabbed in Italy. In about one-third of cases, both in elective and emergency scenarios, this was associated with chest X-Ray, the use of which remained stable with a slight increase in the subsequent phases, accompanied by a progressive reduction in chest CT use, which was initially recommended (19% vs. 7.5% vs. 8.8% in elective surgery and 20.8% vs. 11.5% vs. 13.3% in emergency surgery) (Fig. 7).

## Safety profiles of surgical operations in Italy during the three phases of the pandemic

### PPE

In phase I, for surgical procedures on COVID-19-negative patients, 76.6% of respondents protected themselves with a surgical and 59.3% with an FFP2 mask. Stable percentages were found in the following two phases, with an increase in FFP2 masks (74.3%) in phase III.

Goggles or visors were used by half of the respondents in each of the three phases.

**Fig. 8:**
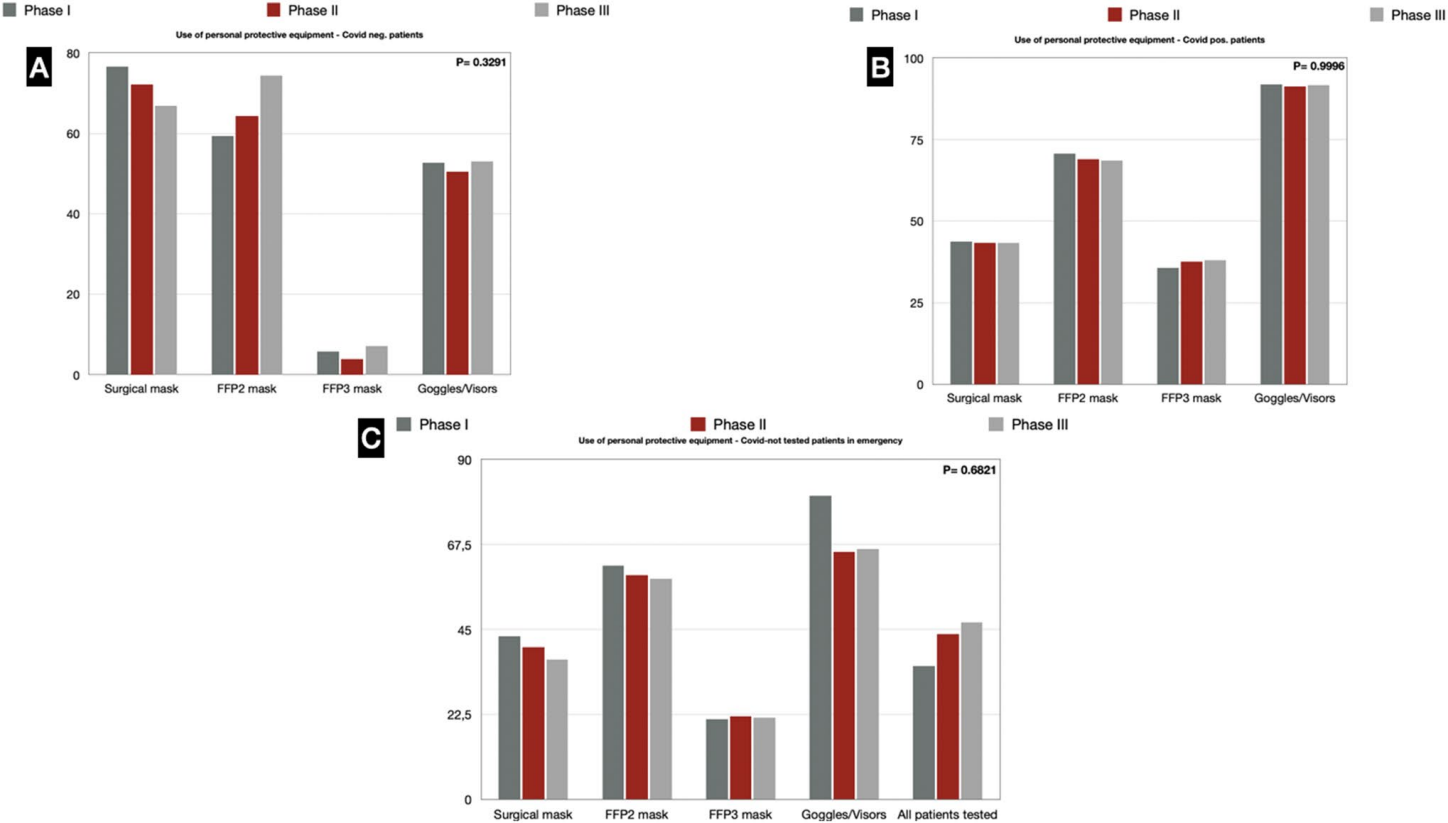
**a** Use of personal protective equipment—COVID-19-negative patients; **b** Use of personal protective equipment—COVID-19-positive patients; **c** Use of personal protective equipment—COVID-19 untested patients in emergency

In the case of surgical procedures on COVID-19-positive patients, most surgeons used an FFP2 mask in all phases (70.8% vs. 69% vs. 68.6%), one-third used an FFP3 mask (35.8% vs. 37.6% vs. 38.1%), and more than 90% always wore goggles or visors (92% vs. 91.2% vs. 91.6%). Similar behavior was found in patients not screened for COVID-19 admitted operated in an emergency setting (Fig. 8) (Supplementary Digital Content_Table5).

### SEDs and HEDs

**Fig. 9:**
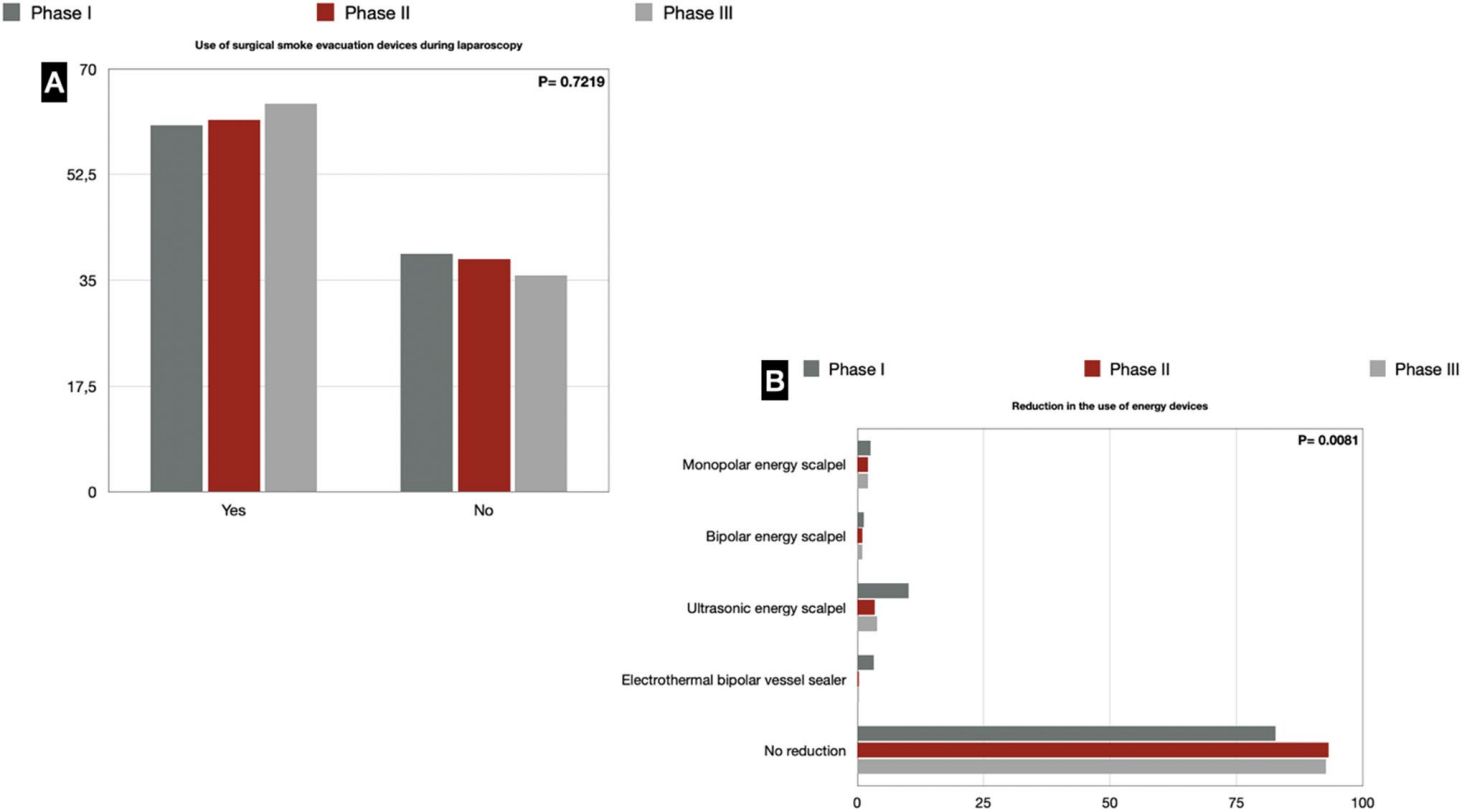
**a** Use of surgical smoke evacuation devices during laparoscopy; **b** Reduction in the use of high-energy devices

Sixty percent of the respondents used SEDs during laparoscopic procedures since phase I, 61.6% adopted them in phase II, and 64.2% in phase III. This means that up to 40% have never used a SED. Across the three periods, commercially available devices were the most used, employed by 49.2% of surgeons. Almost all respondents (82.8% vs. 93.2% vs. 92.7%) in each analyzed pandemic phase did not report a decrease in the use of HEDs (Fig. 9).

## Discussion

This study provides a series of frames of the impact of the COVID-19 pandemic on the Italian surgical community and the response attitudes that surgeons have implemented to face this health crisis. The peculiarity of this survey is that it has taken three photographs of each of the three different phases of the pandemic: the first one, in correspondence with the first global pandemic wave (March–May 2020), the second (June–September 2020) in a period of relative calm in the spread of the virus and a third phase (October–December 2020) which saw the second wave of the pandemic in Italy.

The comparative analysis of what happened in the three phases allowed us to understand how surgeons have changed and evolved their practice, which recommendations have been adopted, and which ones could be modified.

In particular, this survey aimed to analyze three aspects. The first one, concerning the reorganization of surgical departments, the number of operations performed in elective and emergency settings, and the surgical technique used. The second one, regarding the COVID-19 screening methods, intending to improve their availability and reliability. The third aimed to explore how surgeons' attitudes in the operating room had changed: which and when PPEs were used, the use of SEDs for the safe management of the pneumoperitoneum during laparoscopy, and the use of HEDs.

This survey's first significant strength is the high response rate obtained: 50% of the departments invited to participate have signed up with their data. This makes us confident that the respondents reflect the attitudes of the Italian surgical population. A meta-analysis by Shih and Fan showed that the average response rate for email surveys is 33% [13], and a recent report demonstrated that surveys administered on a surgical topic are expected to get a low response rate if given electronically (36.4%) and nationally (42%) [14].

Our response rate vastly exceeds this limit. The answers obtained bring out the Italian surgical departments' reality and offer a realistic and reliable picture of how the surgical community has reacted to the COVID-19 pandemic.

A first important aspect that emerges from the analysis of the responses is that after nine months from the beginning of the pandemic, we are still far from returning to the volumes of surgical activity of the pre-COVID-19 era. Moreover, the survey showed that almost all Italian hospitals had maintained a mixed configuration in managing positive and negative COVID-19 patients. These data contrast with the recommendations to identify hospitals or defined pathways that are COVID-19-free, where it is possible to continue to manage all patients suffering from other diseases who need treatment. For surgical patients, the CovidSurg group demonstrated that postoperative pulmonary complication rates were lower for patients in COVID-19-free surgical pathways during the SARS-CoV-2 pandemic [15].

As already reported by previously published surveys [16, 17], in the first pandemic phase, there was an apparent reduction in the activity of the surgical departments, primarily with the reduction in the number of effective beds dedicated to surgical activity: overall the 59% of surgical departments reduced their beds' availability, and 11.9% of them were closed during the phase I of the pandemic. The goal was to redirect nursing and medical-surgical staff to the management of COVID-19 patients, as demonstrated by the fact that about 50% of the departments had at least one member of their staff referred to the care of COVID-19 non-surgical patients.

It is worth noting that, although there has been an improvement in phase II, with the advent of the second pandemic wave, Italian surgical departments have experienced a return to a scenario that is similar to that of phase I: 7.7% of the surgical departments are still closed in phase III, 55.4% of the respondents declared that they still had fewer than 20 beds assigned to a surgical activity, and the range of 20–30 beds, the most represented in the pre-COVID-19 era (47.6%), remained stably reduced at 30%.

Consequently, in the first and third phases, there was a significant reduction in the elective surgical activity, which means that we are currently still not able to guarantee timely cures for a series of surgical pathologies, including tumors. Regarding emergency surgery, there was an even more significant contraction of the activity. The inevitable collateral damages that the pandemic brings are the diagnostic delays, the increase in the diagnosis-to-treatment interval for neoplastic diseases, and the delay between neoadjuvant treatment and surgery [16, 18].

Since some studies [19, 20] had investigated the possibility of viral aerosolization (HPV, HBV, HIV) by both pneumoperitoneum and surgical smoke during laparoscopic surgery, some had suggested not to use laparoscopy [10, 21, 22], even if there is no evidence that SARS-CoV-2 could spread by aerosolization. As it is well known, laparoscopic surgery represents the best choice of treatment for many disease [23, 24] and the standard rate of laparoscopic procedures in Italy ranges from 35% up to 60–80% in high-trained departments [25–27]. The range of 50–80% of operations performed by laparoscopy in our survey had dropped to 22.3% in the first phase, highlighting that such recommendations had a strong impact during the first pandemic phase.

However, in phases II and III, Italian surgeons have resumed performing laparoscopic surgery more frequently. This is because during phase II, under the pressure of the surgical community, several published articles have reduced the strength of this recommendation [28, 29].

Therefore, the new recommendation is to use laparoscopy when there is a clear benefit for the patient, within a well-trained team, and with the adoption of every protective device, such as PPE and SEDs [30, 31].

The present survey has also highlighted that surgeons have quite understood the need to protect themselves using the necessary PPE with the use of glasses/facial shield and FFP2 or FFP3 masks during the unfolding of the pandemic. In the first phase, there was a shortage in the availability of these PPE [32], but even subsequently the increase in their use was mild and with no statistically significant difference.

Conversely, almost 40% of the respondents have never adopted SEDs in all three phases. This means that the recommendations on the appropriate way of managing the pneumoperitoneum and the surgical plume were not followed appropriately. In this scenario, it is unclear whether laparoscopic smoke represents a greater risk than that created during open surgery [20]. Even in open surgery, there would be a dispersion of surgical plume, which has the same potential to carry viral transmission compared to laparoscopy [33]. Therefore, even in open surgery, devices dedicated to surgical smoke aspiration should be adopted, and it becomes more complex to control rather than inside a closed abdomen [34].

Likewise, little or nothing has been implemented by the advice to reduce the use of specific HEDs, reduce aerosolization of particles, and the potential risk of viral emission. On the contrary, our survey shows that in the development of the three phases, the percentage of those who followed this recommendation was increasingly lower. Italian surgeons have perceived that recommendations against the use of laparoscopy or some HEDs seem to be linked more to theoretical and physio-pathological considerations than to real data [28, 35].

Regarding the screening policies, it is clear that there has been a progressive enhancement of testing the patient before entering the ward or operating room. The percentage of departments that performed molecular tests on a nasopharyngeal swab in phase I was 81% for elective patients and 77.9% for those admitted in an emergency setting. This percentage rose to 87.6% and 81.9%, respectively, in the second phase and even more in the third: 90.7% and 85.4%.

All departments currently perform screening tests on their incoming patients, but it should not be ignored that 10% of responders still do not use the molecular swab test to check patients' negativity. The ACIE Appy Study [36] reported that, during the first pandemic wave, about half of the respondents screened surgical patients only in case of respiratory symptoms and that 12% did not test patients in emergency admission at all. This opens the debate not only on the surgeon's but also on the patient's safety, as the increased rate of postoperative complications for positive asymptomatic patients is well demonstrated [37, 38].

This survey shows that the percentages of positive COVID-19 patients' interventions always remain low both in the elective and emergency settings. Knowing if the patient has contracted a COVID-19 infection is also crucial for deciding to proceed with conservative treatment, where possible, and to postpone surgery [38, 39].

Despite response rate was among the highest for an online survey, the first limitation is that the kind of study does not allow us to gain strong evidences. Furthermore, there are no data collecting of the pre-COVID-19 situation that can be directly compared with the answers obtained. Similarly, the use of ranges instead of absolute numbers gave us a more approximate evaluation and sometimes not very representative data.

## Conclusion

This survey offers an exact and faithful representation of what has been the reality of the Italian surgical departments that have had to face the COVID-19 pandemic. The critical impact that the spread of the SARS-Cov2 has had and is still having on elective and emergency surgical in Italy has been highlighted, with many departments that are still closed or powerfully downsized. Our survey clearly showed that almost all Italian hospitals had maintained a mixed configuration in managing positive and negative COVID-19 patients. These data contrast with the recommendations to identify hospitals or defined pathways that are COVID-19-free, where it is possible to continue to manage all patients suffering from other diseases who need treatment.

Moreover, the recommendations that emerged since the start of the pandemic had a low level of evidence and, in any case, we believe they should have been more accurate and clear. They had little impact on Italian surgeons' behavior in the operating room, who, despite a first phase, did not give up the concrete benefits of laparoscopy, and who largely ignored the advice on using SEDs and HEDs. Further research should analyze the scenario in the upcoming months concerning the pandemic phases' progress and assess if better health policies and more reliable scientific evidence will improve surgery in the time of COVID-19.

## Supplementary Information

Below is the link to the electronic supplementary material.

Electronic supplementary material 1 (DOCX 37 kb)
