# Supplementary material for: Changes in surgicaL behaviOrs dUring the CoviD-19 pandemic. The SICE CLOUD19 Study
Source: Updates Surg. 2021 Mar 3;73(2):731–44. doi: 10.1007/s13304-021-01010-w (PMC7926077; doi:10.1007/s13304-021-01010-w)
Supplement: Supplementary file 1 — Electronic supplementary material 1 (DOCX 37 kb) [file 13304_2021_1010_MOESM1_ESM.docx]

**Update in Surgery**

**Changes in surgicaL behaviOrs dUring the CoviD-19 pandemic. The SICE CLOUD19 Study.**

Umberto Bracale^1^. Mauro Podda^2^. Simone Castiglioni^1.3^. Roberto Peltrini^1^. Emanuele Botteri^4^. Alberto Sartori^5^. Alberto Arezzo^6^. Francesco Corcione^1^. Ferdinando Agresta^7^ and the CLOUD-19 Collaborative Group

1. Department of General Surgery and Specialties. University Federico II of Naples. Italy
2. Department of Emergency Surgery. Policlinico Universitario di Monserrato. Azienda Ospedaliero-Universitari di Cagliari. Cagliari. Italy.
3. Department of Medical. Oral and Biotechnological Sciences. University “G. D’Annunzio” Chieti-Pescara. Italy
4. Department of Surgery. ASST Spedali Civili Brescia. Montichiari. Brescia. Italy.
5. Department of General. Oncological and Metabolic Surgery. Castelfranco and Montebelluna Hospitals. Treviso. Italy.
6. Department of Surgical Sciences. University of Torino. Torino. Italy.
7. Department of General Surgery. Ospedale di Vittorio Veneto. ULSS 2 Marca Trevigiana. Italy.

**Corresponding Author:**

**Name:** Simone

**Surname:** Castiglioni

**Department:** Department of General Surgery and Specialties. University Federico II of Naples. Italy

**Email:** [simone.castiglioni90@gmail.com](mailto:simone.castiglioni90@gmail.com)

**Phone:** 00393402123140

Suppl. Digit. Content. Table 1. Geographical distribution and hospital characteristics of surveyed surgeons

| **Geographical distribution** | Northern regions | 116 (51.2%) | Lombardia | | 52 (23%) |
| --- | --- | --- | --- | --- | --- |
|  |  |  | Piemonte | | 20 (8.8%) |
|  |  |  | Veneto | | 19 (8.4%) |
|  |  |  | Emilia-Romagna | | 15 (6.6%) |
|  |  |  | Friuli Venezia Giulia | | 4 (1.8%) |
|  |  |  | Liguria | | 3 (1.3%) |
|  |  |  | Trentino Alto Adige | | 2 (0.9%) |
|  |  |  | Valle d’Aosta | | 1 (0.4%) |
|  | Central regions | 35 (15.6%) | Lazio | | 11 (4.9%) |
|  |  |  | Abruzzo | | 6 (2.7%) |
|  |  |  | Marche | | 6 (2.7%) |
|  |  |  | Umbria | | 6 (2.7%) |
|  |  |  | Toscana | | 5 (2.2%) |
|  |  |  | Molise | | 1 (0.4%) |
|  | Southern regions | 75 (33.2%) | Campania | | 33 (14.6%) |
|  |  |  | Puglia | | 12 (5.3%) |
|  |  |  | Calabria | | 11 (4.9%) |
|  |  |  | Basilicata | | 7 (3.1%) |
|  |  |  | Sardegna | | 7 (3.1%) |
|  |  |  | Sicilia | | 5 (2.2%) |
| **Hospital characteristics** | Not-teaching Hospital  Teaching Hospital | | | 153 (67.7%)  73 (32.3%) | |
|  | Hub Hospital  Spoke Hospital | | | 132 (58.4%)  94 (41.6%) | |
|  | Public Hospital  Hospital affiliated to NHS  Private Clinic | | | 198 (87.7%)  27 (11.9%)  1 (0.4%) | |

Suppl. Digit. Content. Table 2. Elective and Urgent Surgery Rate across the three phases

| **Procedures** | **Range** | **First Phase (%)** | **Second Phase (%)** | **Third Phase (%)** | **P value** |
| --- | --- | --- | --- | --- | --- |
| **Elective** | < 20 | 29.2 | 10.3 | 18.3 | < 0.00001 |
|  | 20 - 50 | 33.5 | 21.9 | 27.8 |  |
|  | 50 - 100 | 21.9 | 22.3 | 28.2 |  |
|  | 100 - 200 | 13.3 | 24.9 | 15.8 |  |
|  | > 200 | 2.1 | 20.6 | 9.9 |  |
| **Emergency** | < 20 | 43.3 | 27.1 | 36.5 | 0.001843 |
|  | 20 - 50 | 33.0 | 33.0 | 33.9 |  |
|  | 50 - 100 | 17.2 | 24.9 | 21.9 |  |
|  | 100 - 200 | 5.6 | 12.0 | 7.3 |  |
|  | > 200 | 0.9 | 3.0 | 0.4 |  |

Suppl. Digit. Content. Table 3. Rate of laparoscopic adoption in elective and urgent surgery across the three phases

| **Procedures** | **Range** | **First Phase (%)** | **Second phase (%)** | **Third Phase (%)** | **P-value** |
| --- | --- | --- | --- | --- | --- |
| **Elective laparoscopy** | < 20% | 25.8 | 12.4 | 19.3 | 0.000703 |
|  | 20 – 50% | 36.4 | 33.5 | 27.0 |  |
|  | 50 – 80% | 22.3 | 34.8 | 35.2 |  |
|  | > 80% | 15.5 | 19.3 | 18.5 |  |
| **Urgent laparoscopy** | < 20% | 44.6 | 30.9 | 36.1 | 0.037716 |
|  | 20 – 50% | 35.2 | 40.7 | 34.7 |  |
|  | 50 – 80% | 13.3 | 20.2 | 22.3 |  |
|  | > 80% | 6.9 | 8.2 | 6.9 |  |

Suppl. Digit. Content. Table 4. Rate of COVID+ surgeons across the three phases

|  | **Range** | **First Phase (%)** | **Second Phase (%)** | **Third Phase (%)** | **P value** |
| --- | --- | --- | --- | --- | --- |
| **Percentages of COVID+ Surgeons** | < 5% | 74.4 | 88.9 | 68.6 | 0.00002 |
|  | 5 – 10% | 10.6 | 4.9 | 15.9 |  |
|  | 10 – 20% | 8.4 | 4.9 | 8.0 |  |
|  | 20 – 30% | 6.6 | 1.3 | 7.5 |  |

Suppl. Digit. Content. Table 5. Use of Personal Protective Equipment (PPE)

| **Patient** | **Kind of PPE** | **First Phase (%)** | **Second Phase (%)** | **Third Phase (%)** | **P-value** |
| --- | --- | --- | --- | --- | --- |
| **COVID -** | Surgical mask | 76.6 | 72.1 | 66.8 | 0.32912 |
|  | FFP2 | 59.3 | 64.2 | 74.3 |  |
|  | FFP3 | 5.8 | 4.0 | 7.1 |  |
|  | Glasses/Visors | 52.7 | 50.4 | 53.1 |  |
| **COVID +** | Surgical mask | 43.8 | 43.4 | 43.4 | 0.999655 |
|  | FFP2 | 70.8 | 69.0 | 68.6 |  |
|  | FFP3 | 35.8 | 37.6 | 38.1 |  |
|  | Glasses/Visors | 92.0 | 91.2 | 91.6 |  |
| **Not tested in emergency.** | Surgical mask | 43.3 | 40.3 | 37.1 | 0.682168 |
|  | FFP2 | 61.9 | 59.3 | 58.4 |  |
|  | FFP3 | 21.2 | 22.1 | 21.7 |  |
|  | Glasses/Visors | 70.4 | 65.5 | 66.4 |  |
|  | All patient tested | 35.4 | 43.8 | 46.9 |  |

***SURVEY QUESTIONS***

***General organization of the departments***

1. The hospital you work in is:
   - - Teaching hospital
     - Non-teaching hospital
2. The hospital you work in is:
   - - Hub center
     - Spoke center
3. The hospital you work in is:
   - - Public
     - Private
     - Affiliated to National Health System
4. The hospital you work in during Phase I of the pandemic could be defined as:
   - - dedicated exclusively to the care of Covid-positive patients
     - dedicated exclusively to the care of Covid-negative patients
     - mixed care (both Covid-positive and Covid-negative patients)
5. The hospital you work in during Phase II of the pandemic could be defined as:
   - - dedicated exclusively to the care of Covid-positive patients
     - dedicated exclusively to the care of Covid-negative patients
     - mixed care (both Covid-positive and Covid-negative patients)
6. The hospital you work in during Phase III of the pandemic could be defined as:
   - - dedicated exclusively to the care of Covid-positive patients
     - dedicated exclusively to the care of Covid-negative patients
     - mixed care (both Covid-positive and Covid-negative patients)
7. How many beds are assigned to the surgery department where you work?
   - - <20
     - 20-30
     - >30
8. How many effective beds dedicated to surgical activity did the surgery department where you work have during phase I of the pandemic?
   - - <20
     - 20-30
     - >30
     - It has been closed
9. How many effective beds dedicated to surgical activity did the surgery department where you work have during phase II of the pandemic?
   - - <20
     - 20-30
     - >30
     - It has been closed
10. How many effective beds dedicated to surgical activity did the surgery department where you work have during phase III of the pandemic?
    - - <20
      - 20-30
      - >30
      - It has been closed
11. How many elective surgical procedures were performed during Phase I of the pandemic in your department?
    - - <20
      - 20-50
      - 50-100
      - 100-200
      - •> 200
12. How many elective surgical procedures were performed during Phase II of the pandemic in your department?
    - - <20
      - 20-50
      - 50-100
      - 100-200
      - •> 200
13. How many elective surgical procedures were performed during Phase III of the pandemic in your department?
    - - <20
      - 20-50
      - 50-100
      - 100-200
      - •>200
14. Out of the total number of surgical procedures performed in election during phase I of the pandemic in your department. how many were performed with a minimally invasive technique?
    - - <20%
      - 20-50%
      - 51-80%
      - •> 80%
15. Out of the total number of surgical procedures performed in election during phase II of the pandemic in your department. how many were performed with a minimally invasive technique?
    - - <20%
      - 20-50%
      - 51-80%
      - •> 80%
16. Out of the total number of surgical procedures performed in election during phase III of the pandemic in your department. how many were performed with a minimally invasive technique?
    - - <20%
      - 20-50%
      - 51-80%
      - •> 80%
17. How many urgent surgical procedures were performed during Phase I of the pandemic in your department?
    - - <20
      - 20-50
      - 50-100
      - 100-200
      - •> 200
18. How many urgent surgical procedures were performed during Phase II of the pandemic in your department?
    - - <20
      - 20-50
      - 50-100
      - 100-200
      - •> 200
19. How many urgent surgical procedures were performed during Phase III of the pandemic in your department?
    - - <20
      - 20-50
      - 50-100
      - 100-200
      - •> 200
20. Out of the total number of surgical procedures performed in emergency during phase I of the pandemic in your department. how many were performed with a minimally invasive technique?
    - - <20%
      - 20-50%
      - 51-80%
      - •> 80%
21. Out of the total number of surgical procedures performed in emergency during phase II of the pandemic in your department. how many were performed with a minimally invasive technique?
    - - <20%
      - 20-50%
      - 51-80%
      - •> 80%
22. Out of the total number of surgical procedures performed in emergency during phase III of the pandemic in your department. how many were performed with a minimally invasive technique?
    - - <20%
      - 20-50%
      - 51-80%
      - •> 80%
23. How many Covid-19 positive patients were operated in election during Phase I of the pandemic in your department?
    - - <10
      - 10-20
      - 21-30
      - •> 30
24. How many Covid-19 positive patients were operated in election during Phase II of the pandemic in your department?
    - - <10
      - 10-20
      - 21-30
      - •> 30
25. How many Covid-19 positive patients were operated in election during Phase III of the pandemic in your department?
    - - <10
      - 10-20
      - 21-30
      - •> 30
26. How many Covid-19 positive patients were operated in emergency during Phase I of the pandemic in your department?
    - - <10
      - 10-20
      - 21-30
      - •> 30
27. How many Covid-19 positive patients were operated in emergency during Phase II of the pandemic in your department?
    - - <10
      - 10-20
      - 21-30
      - •> 30
28. How many Covid-19 positive patients were operated in emergency during Phase III of the pandemic in your department?
    - - <10
      - 10-20
      - 21-30
      - •> 30
29. In your department. during Phase I of the pandemic. what percentage of medical personnel were referred to other departments dedicated to the care of Covid-19 positive non-surgical patients?
    - - 0%
      - 1-20%
      - 21-50%
      - 51-80%
      - •> 80%
30. In your department. during Phase II of the pandemic. what percentage of medical personnel were referred to other departments dedicated to the care of Covid-19 positive non-surgical patients?
    - - 0%
      - 1-20%
      - 21-50%
      - 51-80%
      - •> 80%
31. In your department. during Phase III of the pandemic. what percentage of medical personnel were referred to other departments dedicated to the care of Covid-19 positive non-surgical patients?
    - - 0%
      - 1-20%
      - 21-50%
      - 51-80%
      - •> 80%
32. In your department. during Phase I of the pandemic. what percentage of medical personnel tested positive for Covid-19?
    - - <5%
      - 5-10%
      - 11-20%
      - 21-30%
33. In your department. during Phase II of the pandemic. what percentage of medical personnel tested positive for Covid-19?
    - - <5%
      - 5-10%
      - 11-20%
      - 21-30%
34. In your department. during Phase III of the pandemic. what percentage of medical personnel tested positive for Covid-19?
    - - <5%
      - 5-10%
      - 11-20%

• 21-30%

***Screening of the patients***

1. What Covid-19 screening test was used in your department for the admission of elective patients during Phase I of the pandemic? (NB: allow multiple answers)

• PCR on nasopharyngeal swab

• Antigenic on nasopharyngeal swab

• Serological on blood sampling

• Serological on lancing device

• Chest X-ray

• Chest ultrasound

• CT of the chest

• No one

1. What Covid-19 screening test was used in your department for the admission of elective patients during Phase II of the pandemic? (NB: allows multiple answers)

• PCR on nasopharyngeal swab

• Antigenic on nasopharyngeal swab

• Serological on blood sampling

• Serological on lancing device

• Chest X-ray

• Chest ultrasound

• CT of the chest

• No one

1. What Covid-19 screening test was used in your department for the admission of elective patients during Phase III of the pandemic? (NB: allows multiple answers)

• PCR on nasopharyngeal swab

• Antigenic on nasopharyngeal swab

• Serological on blood sampling

• Serological on lancing device

• Chest X-ray

• Chest ultrasound

• CT of the chest

• No one

1. What Covid-19 screening test was used in your department for the admission of urgent patients during Phase I of the pandemic? (NB: allows multiple answers)

• PCR on nasopharyngeal swab

• Antigenic on nasopharyngeal swab

• Serological on blood sampling

• Serological on lancing device

• Chest X-ray

• Chest ultrasound

• CT of the chest

• No one

1. What Covid-19 screening test was used in your department for the admission of urgent patients during Phase II of the pandemic? (NB: allows multiple answers)

• PCR on nasopharyngeal swab

• Antigenic on nasopharyngeal swab

• Serological on blood sampling

• Serological on lancing device

• Chest X-ray

• Chest ultrasound

• CT of the chest

• No one

1. What Covid-19 screening test was used in your department for the admission of urgent patients during Phase III of the pandemic? (NB: allows multiple answers)

• PCR on nasopharyngeal swab

• Antigenic on nasopharyngeal swab

• Serological on blood sampling

• Serological on lancing device

• Chest X-ray

• Chest ultrasound

• CT of the chest

• No one

***Safety of surgical procedures***

1. What personal protective equipment (PPE) did the surgeons in your department wear in the operating room during Phase I of the pandemic with Covid-negative patients? (NB: allows multiple answers)

• Surgical mask

• FFP2 mask

• FFP3 mask

• Glasses and/or Facial shield

• No one

1. What personal protective equipment (PPE) did the surgeons in your department wear in the operating room during Phase II of the pandemic with Covid-negative patients? (NB: allows multiple answers)

• Surgical mask

• FFP2 mask

• FFP3 mask

• Glasses and/or Facial shield

• No one

1. What personal protective equipment (PPE) did the surgeons in your department wear in the operating room during Phase III of the pandemic with Covid-negative patients? (NB: allows multiple answers)

• Surgical mask

• FFP2 mask

• FFP3 mask

• Glasses and/or Facial shield

• No one

1. What personal protective equipment (PPE) did the surgeons in your department wear in the operating room during Phase I of the pandemic with Covid-positive patients? (NB: allows multiple answers)

• Surgical mask

• FFP2 mask

• FFP3 mask

• Glasses and/or Facial shield

• No one

1. What personal protective equipment (PPE) did the surgeons in your department wear in the operating room during Phase II of the pandemic with Covid-positive patients? (NB: allows multiple answers)

• Surgical mask

• FFP2 mask

• FFP3 mask

• Glasses and/or Facial shield

• No one

1. What personal protective equipment (PPE) did the surgeons in your department wear in the operating room during Phase III of the pandemic with Covid-positive patients? (NB: allows multiple answers)

• Surgical mask

• FFP2 mask

• FFP3 mask

• Glasses and/or Facial shield

• No one

1. What personal protective equipment (PPE) did the surgeons in your department wear in the operating room during Phase I of the pandemic with not tested patients in an emergency regime? (NB: allows multiple answers)

• Surgical mask

• FFP2 mask

• FFP3 mask

• Glasses and/or Facial shield

• No one

• All operated patients had been tested for Covid-19

1. What personal protective equipment (PPE) did the surgeons in your department wear in the operating room during Phase II of the pandemic with not tested patients in an emergency regime? (NB: allows multiple answers)

• Surgical mask

• FFP2 mask

• FFP3 mask

• Glasses and/or Facial shield

• No one

• All operated patients had been tested for Covid-19

1. What personal protective equipment (PPE) did the surgeons in your department wear in the operating room during Phase III of the pandemic with not tested patients in an emergency regime? (NB: allows multiple answers)

• Surgical mask

• FFP2 mask

• FFP3 mask

• Glasses and/or Facial shield

• No one

• All operated patients had been tested for Covid-19

1. Have surgical smoke evacuation devices been used in your operating room during laparoscopic interventions in Phase I of the pandemic?

• Yes

• No

1. Have surgical smoke evacuation devices been used in your operating room during laparoscopic interventions in Phase II of the pandemic?

• Yes

• No

1. Have surgical smoke evacuation devices been used in your operating room during laparoscopic interventions in Phase III of the pandemic?

• Yes

• No

1. If surgical smoke evacuation devices had been used during laparoscopic interventions during the three phases of the pandemic. which device was used (note: allows multiple responses)

• "Home made" devices

• Airseal

• Olympus UHI-4

• Stryker Pneumoclear

• Erbs IES

• Storz S Pilot

• Ethicon Megadyne

• Medtronic Rapid Vac

• Braun PG 150

• Other

1. Which of the following energy devices listed did you reduce use during Phase I of the pandemic?

• Monopolar electrosurgical unit

• Bipolar electrosurgical unit

• Ultrasound energy devices

• Bipolar electrothermal energy devices

• We have only reduced the use of ultrasonic devices and bipolar electrothermal energy

• We have not reduced the use of any of the devices listed above

1. Which of the following energy devices listed did you reduce use during Phase II of the pandemic?

• Monopolar electrosurgical unit

• Bipolar electrosurgical unit

• Ultrasound energy devices

• Bipolar electrothermal energy devices

• We have only reduced the use of ultrasonic devices and bipolar electrothermal energy

• We have not reduced the use of any of the devices listed above

1. Which of the following energy devices listed did you reduce use during Phase III of the pandemic?

• Monopolar electrosurgical unit

• Bipolar electrosurgical unit

• Ultrasound energy devices

• Bipolar electrothermal energy devices

• We have only reduced the use of ultrasonic devices and bipolar electrothermal energy

• We have not reduced the use of any of the devices listed above
